# Sex- and age-specific trends in mortality from suicide and undetermined death in Germany 1991–2002

**DOI:** 10.1186/1471-2458-5-61

**Published:** 2005-06-06

**Authors:** Jens J Baumert, Natalia Erazo, Karl-Heinz Ladwig

**Affiliations:** 1 Institute of Epidemiology, GSF National Research Center for Environment and Health, Ingolstaedter Landstr. 1, 85764 Neuherberg, Germany; 2 Department for Psychosomatic Medicine, Psychotherapy und Medical Psychology, University Hospital of the Technical University of Munich, Langerstr. 3, 81675 München, Germany

## Abstract

**Background:**

Over the last decade, significant downward linear time trends in suicide mortality were observed in most Western countries. To date, it is not established whether those favourable time trends developed homogeneously for sex and age groups and how they were affected by the number of undetermined deaths.

**Methods:**

Data on suicide mortality and undetermined death from 1991 to 2002 in Germany were obtained from the German Federal Statistical Office. For each year, the age-standardised suicide rate (SR), undetermined death rate (UDR) and total rate (SR+UDR) was calculated by direct standardisation separately for men and women. Time trends were analyzed by Poisson regression estimating the average annual percentage change (AAPC) of the rates for sex and four age groups (15–24, 25–44, 45–74, ≥ 75 years).

**Results:**

A significant decline of the SR was observed in all age groups but was less pronounced among the younger ages, particularly among men aged 15–24 years (AAPC -0.7%, p = 0.041). The SR in the oldest male age group (≥ 75 years) declined much stronger (AAPC -3.5%, p < 0.001). In women, the AAPC of the SR ranged from -1.7% to -4.6%. The average annual percentage changes in the age groups 25 – 74 years did not differ substantially for SR and SR+UDR. In contrast, due to an increase of undetermined deaths for subjects ≥ 75 years, time trends in this age group were affected by the number of undetermined deaths, especially in women.

**Conclusion:**

Observing downward trends in suicide mortality with lower declines for younger subjects, prevention strategies should focus in particular on younger subjects.

## Background

Over the last two decades, significant downward trends in overall suicide rates have been observed in North America and in most countries of Europe [1-4]. Chishti et al. on behalf of the EUROSAVE working group [1] analysed data from 15 countries in the European Union (EU) and revealed downward trends in most EU countries except Ireland and Spain.

However, reasons for those favourable trends have not been ascertained so far. Moreover, it is not established whether those favourable time trends developed homogeneously for sex and age groups. It is not unlikely that the general decrease of suicide mortality may hide rises in certain age and sex groups. Guaiana et al. analysed age- and sex-specific suicide rates in Italy from 1986 to 1996 and found that in men and women over 45 years of age, rates progressively decreased while in men between 15 and 44 years of age, suicide rates progressively rose [5]. Gunnell and Middleton [6] revealed an overall decline of age-standardised suicide rates in England and Wales by 18% between 1981 and 1998. However, an increase of suicide rates for men aged 15–44 years was embedded in the downward trend.

Inconsistencies of death certification practices and the changes in the estimated number of unknown cases may bias the accuracy of national suicide figures. Therefore, analyses of suicide trends often include "undetermined deaths" in their calculations to take account of misclassifications. Chishti *et al*. [1] negated misclassification as reason for changing suicide rates for most EU member states, however, showed borderline significant upward linear trends in death due to undetermined causes for Belgium for the years 1994–1995 and Germany for the years 1984 – 1997.

The aim of the present study was to investigate whether time trends of suicide mortality in Germany over the 12-year observation period from 1991 to 2002 developed homogeneously for men and women older than ≥ 15 years in four age groups. Another major issue of the present study was a sex- and age-specific investigation whether trends of deaths due to undetermined causes affected suicide mortality trends.

## Methods

Mortality data were provided by the Federal Statistical Office in Germany and allowed to calculate suicide and undetermined death rates for each year, standardised by sex and age [7]. Death by suicide was defined as "intentional self-harm" according to the ICD-9 (International Classification of Diseases, 9th revision; WHO, 1977) categories E950 to E959 in the time period from 1991 to 1997 and to the ICD-10 (10th revision; WHO, 1992) categories X60 to X84 and Y87 from 1998 to 2002 [8,9]. Deaths due to undetermined causes were defined according to ICD-9 (E980-E989) and ICD-10 (Y10-Y34, Y89).

Based on these data, three different "event rates" were defined: Suicide rate (SR), undetermined death rate (UDR) and the total rate (SR+UDR) which included deaths by suicide and undetermined causes. To adjust these event rates for annual changing population figures and age structures, direct standardization with 10-year age interval groups (15–24, ..., ≥ 75 years) were used to estimate age-adjusted event rates for each sex [10]. The population of Germany in 1991 was chosen as standard population. For four age groups (15–24, 25–44, 45–74, ≥ 75 years), event rates were estimated for 100,000 inhabitants aged ≥ 15 years and per year leading to a data set with 96 observations (12 years per two sexes per four age groups).

Time trends in the 12-year observation period from 1991 to 2002 for the events suicide and undetermined death as well as for the event suicide or undetermined death were analyzed by Poisson regression models using the event rate under concern as outcome variable [11]. Estimating the regression equation

Log (event rate) = α + β * YEAR + ε

(containing YEAR as the year of event as linear term), the average annual percentage change (AAPC) of the event rate was given by

AAPC = [exp() - 1] * 100

and calculated separately for each sex and age group (four categories).

Modifications of time trends of the age-standardized suicide, undetermined and total rate by sex were tested by additionally including variables sex and the interaction term year of event * sex in the regression equation:

Log (event rate) = α + β_1 _* YEAR

+ β_2 _* SEX

+ γ * YEAR * SEX + ε

A rejection of the test hypotheses γ = 0 indicates a significant modification of time trends by sex. Moreover, for each sex, modifications of time trends by age group was tested by including variables age group with interaction terms age group*year of event in the regression equation:

Log (event rate) = α + β_1 _* YEAR

+ β_2 _* AGE(25–44) + β_3 _* AGE(45–74) + β_4 _* AGE(≥ 75)

+ γ_1 _* YEAR * AGE(25–44) + γ_2 _* YEAR * AGE(45–74)

+ γ_3 _* YEAR * AGE(≥ 75) + ε

A rejection of the test hypotheses γ_1 _= 0, γ_2 _= 0, γ_3 _= 0 indicates a significant modification of time trends by age group.

To correct for over-dispersion of the Poisson model, the dispersion parameter was estimated by the ratio of the deviance to its associated degrees of freedom [11].

For all statistical analyses, a P value less than 0.05 was considered to be statistically significant. All analyses were performed with the statistical software package SAS for Unix, Version 8.2 [12].

## Results

### Overall time trend

**Figure 1:**
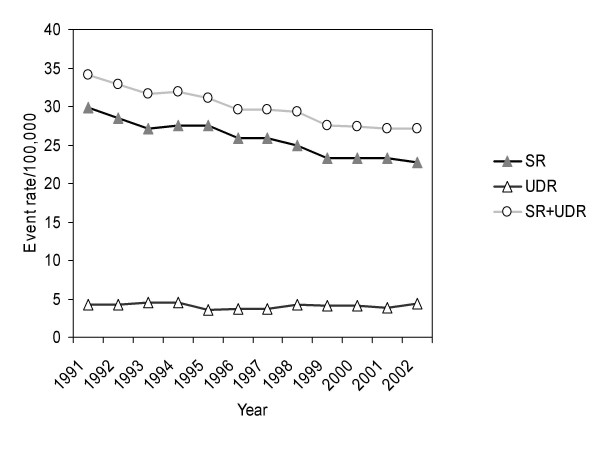
Age-standardized suicide rate (SR), undetermined death rate (UDR) and total rate (SR+UDR) from 1991 to 2002 for men aged ≥ 15 years.

**Figure 2:**
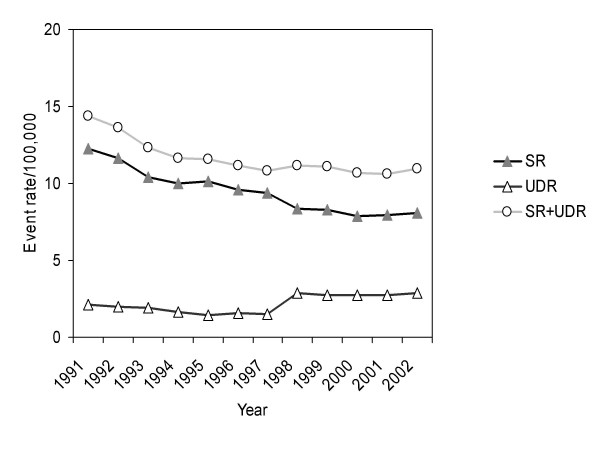
Age-standardized suicide rate (SR), undetermined death rate (UDR) and total rate (SR+UDR) from 1991 to 2002 for women aged ≥ 15 years.

From 1991 until 2002, a total of 146 709 completed suicides for subjects aged ≥ 15 years were recorded (104 675 men, 41 404 women). During this 12-year observation period, a decline of the age-standardised suicide rate (SR) was observed from 29.80 in 1991 to 22.78 in 2002 for men (Figure 1) and from 12.25 in 1991 to 8.09 in 2002 for women (Figure 2). The percentage change of the suicide rate was -23.6% in men and -34.0% in women leading to an impaired men-to-women-ratio in suicide rate which changed from 2.4 (1991) to 2.8 (2002). On average, age-standardised suicide rates declined significantly per year in men (AAPC -2.4%, 95% CI -4.1 to -0.7) and women (AAPC -4.0%, 95% CI -6.6 to -1.4). The downward trend was significantly stronger for women than for men (p < 0.001).

A number of 26 121 events (16 632 men, 9489 women) were classified as undetermined death. No substantial change of the age-standardised undetermined death rate (UDR) during the observation period was observed for men. In contrast, the UDR increased remarkably in women from 2.10 in 1991 to 2.86 in (2002) caused mainly by a strong "leap" from 1997 (with use of ICD-9) to 1998 (with use of ICD-10). Therefore, the total rate SR+UDR ran approximately parallel with SR in men but not in women.

### Age-specific time trends

The decline in suicide rates was observed in all age groups but was less pronounced among the younger ages, particularly among men aged 15 – 24 years with an AAPC of -0.7 (95% CI: -1.3 to 0.0, p = 0.041) (Table 1). Compared to these, the suicide rate in the oldest male age group (≥ 75 years) declined by 3.5-times. In women, the modification of trends by age group was similar and the downward trend in each age interval was markedly higher compared to men (Table 2). The SR time trends were significantly modified by age group in men (p < 0.001) and women (p = 0.005).

**Table 1:** Average annual percentage changes (AAPC) of the suicide rate (SR), undetermined death rate (UDR) and total rate (SR+UDR) in men aged ≥ 15 years over a 12-year observation period in Germany, 1991–2002

Men	***1991–1997***		***1998–2002***		***1991–2002***	
	AAPC (95% CI)	P value	AAPC (95% CI)	P value	AAPC (95% CI)	P value
**SR**						
15–24 years	-0.9 (-2.7 – 1.0)	0.361	-0.3 (-1.5 – 0.9)	0.596	-0.7 (-1.3 – 0.0)	0.041
25–44 years	-1.5 (-2.5 – -0.5)	0.002	-2.1 (-3.3 – -0.9)	0.001	-2.2 (-2.6 – -1.8)	<.001
45–74 years	-2.5 (-3.5 – -1.5)	<.001	-1.4 (-3.3 – 0.4)	0.135	-2.2 (-2.7 – -1.8)	<.001
≥ 75 years	-2.6 **(**-3.9 – -1.2)	<.001	-2.3 (-4.2 – -0.3)	0.024	-3.5 (-4.2 – -2.9)	<.001
p value for interaction*		0.395		0.596		<.001
**UDR**						
15–24 years	-1.9 (-4.2 – 0.3)	0.095	-3.0 (-9.1 – 3.4)	0.350	-0.6 (-2.0 – 0.7)	0.353
25–44 years	-1.4 (-3.7 – 1.0)	0.245	-2.9 (-6.4 – 0.7)	0.109	-2.7 (-3.7 – -1.7)	<.001
45–74 years	-4.9 (-8.0 – -1.6)	0.004	3.1 (-0.9 – 7.3)	0.125	-0.5 (-2.3 – 1.4)	0.620
≥ 75 years	-5.8 (-10.1 – -1.2)	0.014	-0.1 (-4.0 – 3.8)	0.945	7.3 (2.9 – 11.9)	0.001
p value for interaction*		0.248		0.367		<.001
**SR+UDR**						
15–24 years	-1.0 (-2.4 – 0.3)	0.136	-0.8 (-2.4 – 0.9)	0.369	-0.6 (-1.2 – -0.1)	0.013
25–44 years	-1.5 (-2.2 – -0.8)	<.001	-2.2 (-3.0 – -1.4)	<.001	-2.3 (-2.6 – -1.9)	<.001
45–74 years	-2.8 (-3.6 – -1.9)	<.001	-0.8 (-2.6 – 1.0)	0.371	-2.0 (-2.5 – -1.6)	<.001
≥ 75 years	-2.9 (-4.1 – -1.7)	<.001	-1.9 (-4.2 – 0.5)	0.117	-2.1 (-2.7 – -1.6)	<.001
p value for interaction*		0.118		0.763		0.009

* p value for test for interaction year of event * age group

Overall sex-specific time trends of the total rate (SR+UDR) revealed significant declines with an AAPC of -2.1 (95% CI: -2.4 to -1.9) in men and -2.4 (95% CI: -3.2 to -1.5) in women. The trends for undetermined death by age groups revealed significant increases of the rates in both sexes for subjects ≥ 75 years leading to a dilution of the stronger decline of the total rate SR+UDR in men aged ≥ 75 years (AAPC -2.1, p < 0.001). For women in the same age group, no decline could be observed for the total rate SR+UDR (AAPC 0.6, p = 0.597). The average annual percentage changes in the other age groups did not differ substantially for SR and SR+UDR.

## Discussion

The present study provides an analysis of trends in suicide mortality and undetermined death rates over a recent 12-years observation period (1991–2002) in Germany and confirms the general downward trend for suicide mortality which reached a change of -23.6% for men and -34.0% for women from 1991 to 2002. These trends were not affected by deaths of undetermined causes in men and women aged 15 to 74 years.

Similar results for time trends of suicide rates were found in other studies. Levi et al. [2] analyzed trends in mortality from suicide excluding undetermined death over a much longer term period of 1965 to 1999 in 47 countries by applying the WHO database and revealed that age-standardized mortality from suicide in most European countries peaked in 1980–84 but decreased thereafter until 1995–98. On average, the downward trend was more pronounced in women compared to men. A similar finding was provided by Chishti et al. (2003) who found significant downward linear time trends in suicide mortality in most European countries for the years 1984 – 1998 [1]. In England and Wales, an overall decline of age-standardised suicide rates in men and women by 18% between 1981 and 1998 was observed [6]. Other studies, analysing data from Italy, Spain, Belgium, and Canada, also showed peaks of prevalence in suicide mortality in the 8^th ^decade of the last century [5,13-15]. In contrast, Stark et al. observed increases in suicide mortality including undetermined deaths for all male age groups in Scotland in 1981 – 1999 while for women a downward trend was confirmed over the same observation period [16].

The present study adds new findings to time trends in suicide mortality and undetermined death by analysing sex- and age-specific developments. In brief, the analysis revealed more pronounced downward trends in women compared to men in all age groups even. Downward suicide mortality trends were less pronounced in age groups 15 – 24 years. This is in agreement with all other studies analysing age effects on suicide rates [5,13-15] except for Greece where increasing suicide trends 1980–1995 were most pronounced in the 45–54 male age group [16]. It is of major concern that especially young people had the least advantage from the general downward trend. In contrast to Gunnell and Middleton [6] who revealed an increase of suicide rates for men aged 15 – 44 years in England and Wales from 1981 to 1998, we found no "hidden" upward trends in particular age groups in our data base. To further maintain and stabilize the favourable downward trend and to tailor preventive measures according to actual requirements, further research highlighting possible causal explanations is urgently required. National prevention strategies should set a particular focus on younger, predominately male subjects.

To date, possible causal explanations remain highly speculative. However, among reasons which may account for the favourable findings in particular among the elderly and the much less favourable findings in the younger age groups are recent improvements in anti-depressive treatment. Recently, Gunnell and Ashby [18] studied associations of antidepressant prescribing and suicide rates in Britain. They revealed an increasing antidepressant prescribing in the elderly from 1991 to 1998 which was associated with a reduction in suicide rate in the observation period [18] Similar results were found in two other studies [19,20]. At the same time, palliative care has substantially improved over the last decade which may have contributed to findings which showed that a diagnosis of cancer in more recent data sets was no longer associated with increased risk of suicide [21,22]. A study investigating trends in risk factors for suicides revealed unfavourable trends with rises in divorce, declines in marriage and increases of unemployment and income inequality which are more related to suicides in younger subjects, especially in men, than in older subjects [23-25].

Informational value of the analysis is primarily restricted to Germany. However, results may be generalized because Germany holds a medial position in average suicide rates among western highly industrialized countries [1]. The time trend analysis was restricted to the last available data base with a 12-years observation period which may be considered as short to identify recently changing time patterns. However, number of cases included by each year was sufficient enough to allow valid calculations. Moreover, we restricted the data set to this time period because substantial changes in political and social structure had occurred beforehand in Germany.

## Conclusion

In the present study, time trends of the suicide rate (SR) and the total rate (SR+UDR) ran similar over a 10-years observation period in the age groups 15 – 74 years indicating that the assessment of suicide mortality in these age groups was not affected by the number of undetermined deaths. Undetermined deaths are mainly considered as probable suicides and were included in several suicide investigations in the past. A study by Ohberg and Lonnquist estimated that almost 9 from 10 undetermined suicides can be regarded as suicides [26]. However, the assessment of time trends in the oldest age group (≥ 75 years) revealed to be rather difficult due to different AAPCs for the suicide rate and the total rate, especially in women. The rate of undetermined deaths increased for both sexes in the oldest age group. Further research is urgently recommended to ascertain reasons for the "leap" of the number of undetermined deaths from 1997 to 1998 for women aged ≥ 75 years.

## List of abbreviations

AAPC Average annual percentage change

SR Suicide rate

SR+UDR Total rate

UDR Undetermined death rate

## Competing interests

The author(s) declare that they have no competing interests.

## Authors' contributions

JJB performed the statistical analysis of the data, contributed to the design of the study and interpretation of the results and drafted the paper. NE contributed to the design of the study and the interpretation of the results. KHL had the idea of the study, contributed to the interpretation of the paper and revisited the paper critically for important intellectual content. All authors read and approved the final draft of the paper.

**Table 2:** Average annual percentage changes (AAPC) of the suicide rate (SR), undetermined death rate (UDR) and total rate (SR+UDR) in women aged ≥ 15 years over a 12-year observation period in Germany, 1991–2002

Women	***1991–1997***		***1998–2002***		***1991–2002***	
	AAPC (95% CI)	P value	AAPC (95% CI)	P value	AAPC (95% CI)	P value
**SR**						
15–24 years	0.2 (-2.7 – 3.1)	0.906	-3.7 (-8.3 – 1.1)	0.131	-1.7 (-3.0 -0.4)	0.012
25–44 years	-2.7 (-4.7 – -0.7)	0.007	-1.3 (-4.2 – 1.6)	0.372	-2.9 (-3.7 -2.1)	<.001
45–74 years	-4.6 (-5.4 – -3.7)	<.001	-1.2 (-1.8 – -0.5)	<.001	-4.2 (-4.7 -3.6)	<.001
≥ 75 years	-5.7 (-7.8 – -3.4)	<.001	0.5 (-1.8 – 2.8)	0.682	-4.6 (-5.8 -3.5)	<.001
p value for interaction*		0.013		0.255		0.005
**UDR**						
15–24 years	-1.7 (-7.1 – 4.1)	0.559	-6.2 (-21.5 – 12)	0.478	-0.2 (-3.5 – 3.2)	0.913
25–44 years	-5.6 (-8.1 – -3.0)	<.001	-2.5 (-7.6 – 2.9)	0.358	-3.1 (-4.5 – -1.7)	<.001
45–74 years	-6.0 (-8.9 – -3.0)	0.001	-1.0 (-3.5 – 1.7)	0.467	-1.9 (-3.5 – -0.2)	0.030
≥ 75 years	-8.6 (-12.1 – -5.0)	<.001	1.6 (-0.4 – 3.7)	0.116	14.7 (7.6 – 22.3)	<.001
p value for interaction*		0.144		0.399		<.001
**SR+UDR**						
15–24 years	-0.2 (-2.2 – 1.9)	0.865	-4.3 (-8.4 – 0.1)	0.053	-1.4 (-2.4 – -0.3)	0.011
25–44 years	-3.2 (-5.0 – -1.3)	0.001	-1.5 (-4.6 – 1.7)	0.361	-3.0 (-3.7 – -2.2)	<.001
45–74 years	-4.7 (-5.5 – -3.9)	<.001	-1.1 (-1.8 – -0.5)	0.001	-3.9 (-4.5 – -3.4)	<.001
≥ 75 years	-6.2 (-8.1 – -4.2)	<.001	1.0 (-0.3 – 2.3)	0.125	0.6 (-1.6 – 2.8)	0.597
p value for interaction*		0.002		0.016		<.001

* p value for test for interaction year of event * age group

## Pre-publication history

The pre-publication history for this paper can be accessed here:


